# An integrated evaluation approach of wearable lower limb exoskeletons for human performance augmentation

**DOI:** 10.1038/s41598-023-29887-0

**Published:** 2023-03-14

**Authors:** Xiao Zhang, Xue Chen, Bo Huo, Chenglin Liu, Xiaorong Zhu, Yuanyuan Zu, Xiliang Wang, Xiao Chen, Qing Sun

**Affiliations:** 1 Systems Engineering Institute, Academy of Military Science, Beijing, 100091 People’s Republic of China; 2 Department of Mechanics, School of Aerospace Engineering, Beijing Institute of Technology, No. 5 South Zhongguancun Street, Beijing, 100081 People’s Republic of China; 3 Institute of Artificial Intelligence in Sports, Capital University of Physical Education and Sports, Beijing, People’s Republic of China; 4 Beijing Institute of Precision Mechatronics and Controls, Beijing, People’s Republic of China

**Keywords:** Metabolomics, Electrical and electronic engineering, Biomedical engineering

## Abstract

Wearable robots have been growing exponentially during the past years and it is crucial to quantify the performance effectiveness and to convert them into practical benchmarks. Although there exist some common metrics such as metabolic cost, many other characteristics still needs to be presented and demonstrated. In this study, we developed an integrated evaluation (IE) approach of wearable exoskeletons of lower limb focusing on human performance augmentation. We proposed a novel classification of trial tasks closely related to exoskeleton functions, which were divided into three categories, namely, basic trial at the preliminary phase, semi-reality trial at the intermediate phase, and reality trial at the advanced phase. In the present study, the IE approach has been exercised with a subject who wore an active power-assisted knee (APAK) exoskeleton with three types of trial tasks, including walking on a treadmill at a certain angle, walking up and down on three-step stairs, and ascending in 11-storey stairs. Three wearable conditions were carried out in each trial task, i.e. with unpowered exoskeleton, with powered exoskeleton, and without the exoskeleton. Nine performance indicators (PIs) for evaluating performance effectiveness were adopted basing on three aspects of goal-level, task-based kinematics, and human–robot interactions. Results indicated that compared with other conditions, the powered APAK exoskeleton make generally lesser heart rate (HR), Metabolic equivalent (METs), biceps femoris (BF) and rectus femoris (RF) muscles activation of the subject at the preliminary phase and intermediate phase, however, with minimal performance augmentation at advanced phase, suggesting that the APAK exoskeleton is not suitable for marketing and should be further improved. In the future, continuous iterative optimization for the IE approach may help the robot community to attain a comprehensive benchmarking methodology for robot-assisted locomotion more efficiently.

## Introduction

Wearable exoskeleton robots are showing great potential in industrial, medical, and military training fields^1–3^. The increasingly concerned lower limb exoskeletons are widely used for augmenting human performance, such as load carrying, walking aid, rehabilitation, and body support^4–6^. According to the power-assisted effects of wearable exoskeleton robots, they can be subdivided into passive and active types. The function of passive type of exoskeletons is mainly to transfer the payload weight to the ground (not to the wearer) and follow the human body motion. Meanwhile, active type exoskeletons treat human body as a load and help to reduce the energy consumption of human body^7^. Although the potential of wearable robotics technology is indisputable, the well-recognized standard has not been formally available so far.

From the initial discussions in a roundtable workshop that was held in December 2014^8^, the specialists have realized the demand for a standardized way to evaluate wearable exoskeleton robots. Subsequently, the robotics community has an increasing interest in scientifically assessing and comparing the performance of exoskeletons by making a standard^9,10^.

Currently, the main way to compare exoskeletons is to hold competitions such as Cybathlon^11^. The main disadvantage of competitions is that scores are usually based on very simple indicators, such as task completion or completion time, which are difficult to reflect multiple aspects of exoskeleton power-assisted effectiveness.

Numerous research on wearable robotics are focused on the development of a particular exoskeleton and testing of the design by measuring metabolic cost or EMG signals, which is commonly conducted by performing simple motions or by walking on a treadmill. The use of a treadmill enables the subject to be in a fixed physical location, thereby allowing for the use of biometrics for evaluation purposes, such as VO_2_, HR, and EMG^12–15^. In addition, electronic-skin (E-skin) based on flexible sensors has been investigated extensively with the goal of providing tactile sensing capability for robots, a generic method for real-time detection of unstable robotic grasping was proposed by Huang et al.^16^. However, few studies have proposed essential PIs from goal-level, task-based kinematics and human–robot interactions to comprehensively assessed the effectiveness of the lower-limb exoskeleton, and few have classified trial tasks scenarios for testing the lower-limb exoskeleton.

Different application fields have various goals in terms of determining the value of a system. We aim to develop an integrated evaluation (IE) approach for lower limb wearable exoskeletons to be used in the military and industrial fields. Two important research questions must be addressed when formulating the evaluation approach for exoskeleton performance: first, which trial are considered when evaluating the functionality of a lower limb exoskeleton? Second, what variables and metrics are used to characterize the performance?

In the present study, we developed an IE approach for the performance augmentation of lower limb exoskeleton. In this approach, the classification of three trial tasks with increasing difficulty or more closely relating to exoskeleton functions is proposed, and nine essential performance indicators (PIs) from goal-level, task-based kinematics, and human–robot interactions are presented. The IE approach prototypes have been exercised with a subject wearing the active power-assisted knee (APAK) exoskeleton to evaluate performance effectiveness.

## Methods

### IE approach of lower limb exoskeletons for performance augmentation

**Figure 1:**
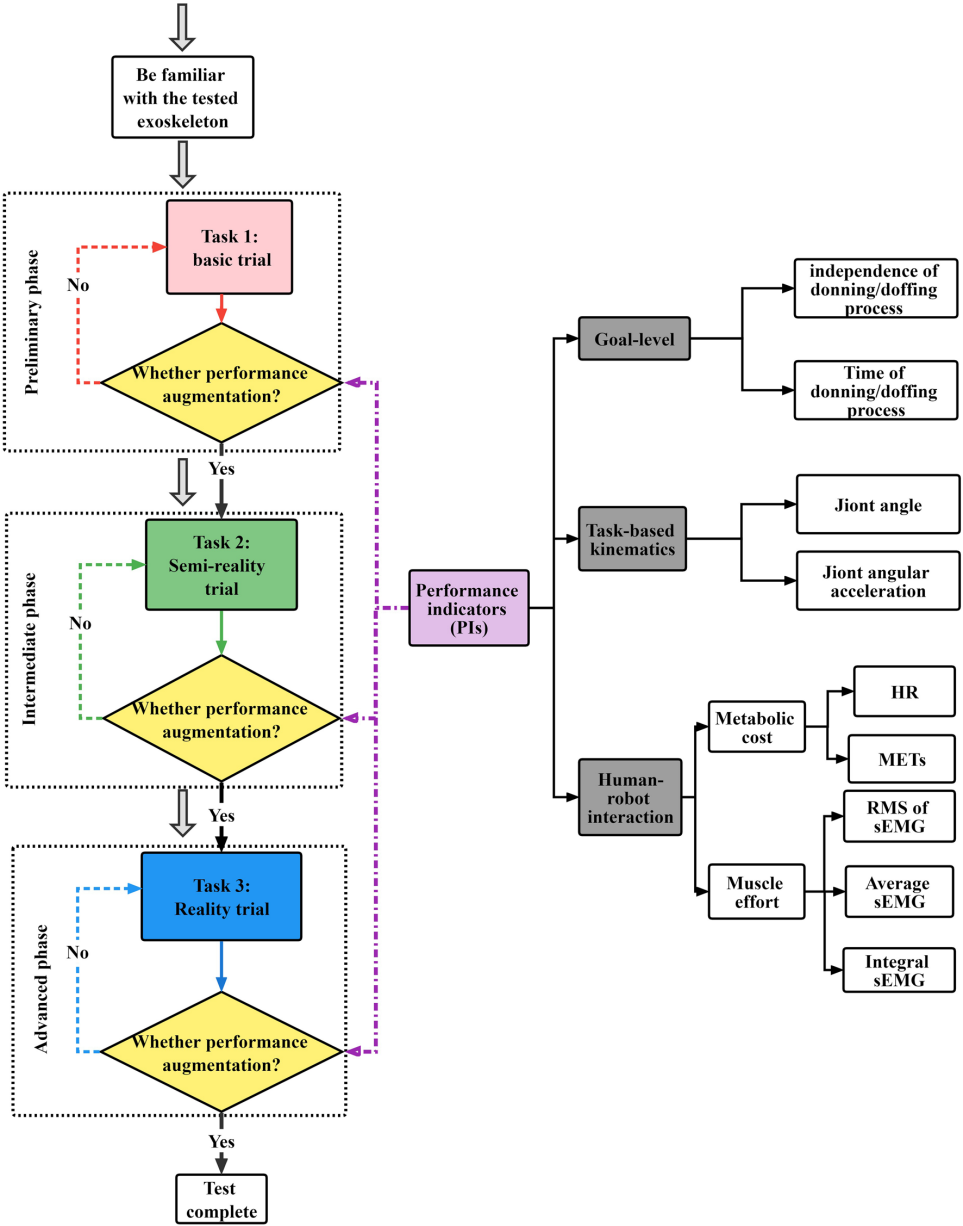
Flow diagram of the IE approach to evaluate the overall exoskeleton performance. Three trial tasks at the preliminary, intermediate, and advanced phases are shown in the dashed box. PIs are divided into three aspects, which are shown in the gray filled box.

Performance augmentation focuses on the healthy person who can perform tasks in some capacity without the exoskeleton. The proposed IE approach associated with a series of essential PIs for power assistance of lower limb exoskeletons includes the following procedures (Fig. 1):

(1) Be familiar with the exoskeleton. Subjects should know the measured exoskeleton to some extent^17^. A practice trial is allowed to familiarize the subjects with the tested exoskeleton and achieve the best man–machine compatibility before the formal measurement trial tasks begin.

(2) Formulate appropriate measurement trial tasks. According to the functionality of the exoskeleton, three phases of performance effectiveness trial tasks from preliminary to advanced are proposed.

#### Preliminary phase I

An experimental basic trial task that is a simplified version of power-assisted scenarios in laboratory for the tested exoskeleton was suggested. At this phase, one or two simple trial tasks, such as walking on level or using a treadmill at certain degree, were mainly conducted to imitate people who are walking on flat ground or slopes in real world.

#### Intermediate phase II

Compared with the trial tasks in the preliminary phase, one or two semi-reality trial tasks with slightly complicated terrains were developed. In this phase, an indoor irregular terrain should be constructed to evaluate the tested abilities of the exoskeleton for transiting between activities.

#### Advanced phase III

Reality trial tasks that are closely related to daily activities, such as ascending stairs in buildings and climbing mountains, can be adopted for the power-assisted exoskeleton. In this phase, task-based kinematics data should be determined by using portable motion capture system instead of optical motion capture system used for routine laboratory tests.

The lower limb exoskeleton tested by the IE approach should satisfy the basic requirements of stability and safety. In other words, some basic testing trials have been completed to guarantee that the exoskeleton does not restrict any basic movements of the subject.

(3) Essential PIs. In present work, PIs are clustered into goal-level variables, task-based kinematics variables, and human–robot interaction variables to quantify performance effectiveness of the tested exoskeleton comprehensively.

#### Goal-level variables

Considering that the tested exoskeleton is completing the preliminary test and some basic indicators were satisfied, the subject is healthy able-bodied. Therefore, the independence and time of donning and doffing processes for the tested exoskeleton are two critical metrics that could directly reflect the ease of donning or doffing processes and user-friendly experience. These two usability aspects were also proposed previously^18,19^.

#### Task-based kinematic variables

Among the kinematic variables, joint angle and joint angular acceleration were considered more important than other indicators in characterizing the kinematic state. The changes in the rotation angle during the whole testing process can be determined by joint angle variable, and the power-assisted feedback and motion posture can be directly featured by joint angular acceleration variable. The kinetics variables are not used to characterize subject motions because these variables cannot be directly measured by using experimental devices and must be simulated using the ground reaction forces as input value, which may result in inaccuracy.

#### Human–robot interaction variables

The most commonly used metabolic cost and muscle effort are used for human–robot interactions^20–25^. The MET, which is a physiological measure that expresses the energy cost of physical activities^26^, and HR are proposed in the metabolic cost section. Muscle effort is generally assessed by measuring the surface electromyographic (sEMG) activity of antagonist pair muscles. The RMS of sEMG, AEMG, and IEMG are recommended to be the main performance metrics for muscle effort.

### Experimental validation

#### Profile testing exoskeleton

**Figure 2:**
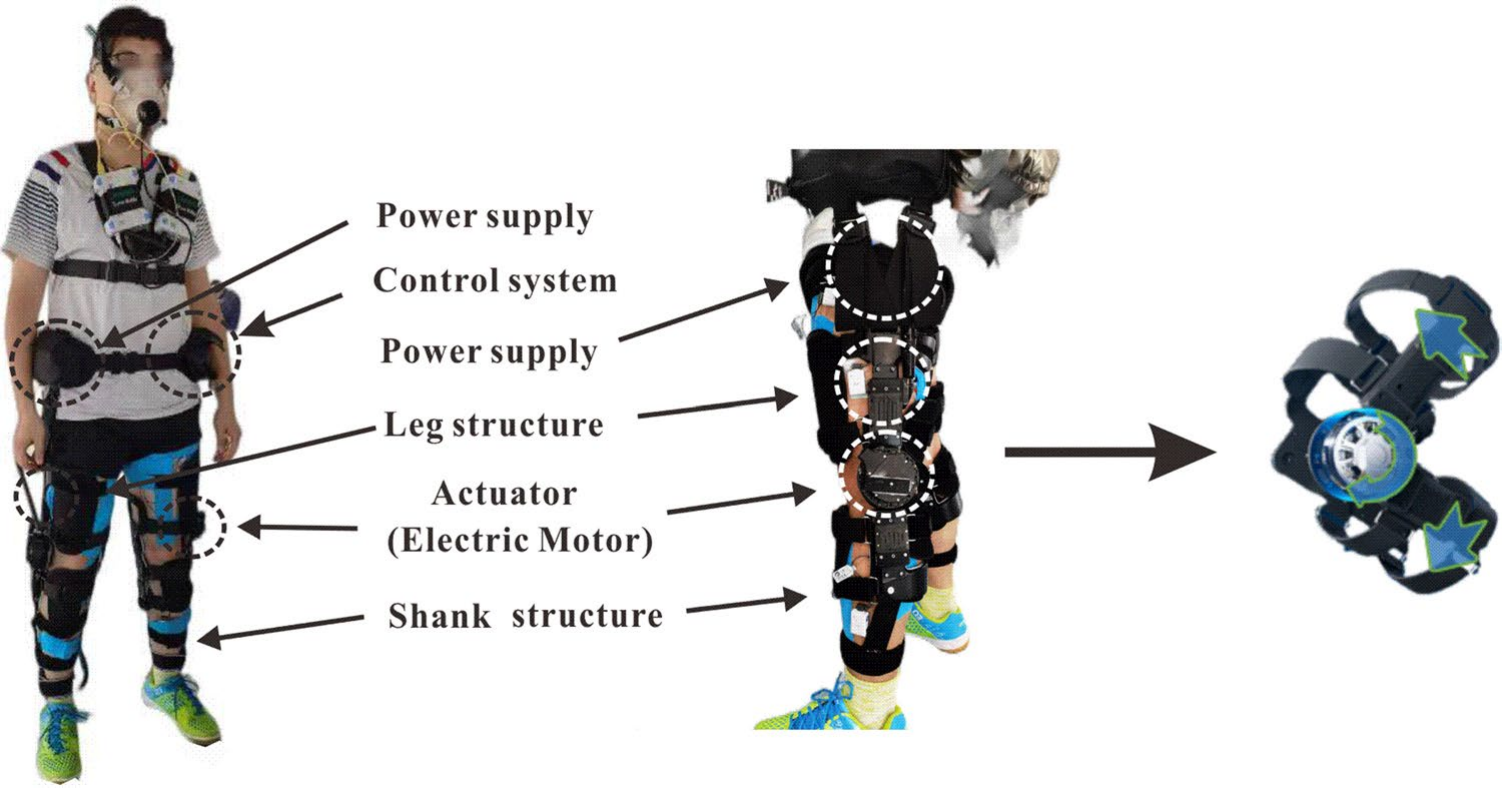
Mechanical design of the APAK exoskeleton.

The APAK exoskeleton developed by our laboratory is composed of a waist belt, a controller, a power supply, a hip flexible strap, a leg structure, and an actuator (electric motor), and its overall weight is 4.4 kg (Fig. 2). The controller and power supply, which were integrated on the waist belt, were connected with the leg structure and the actuator through an electrical cable so that the power supply and signal transmission could be achieved by this electrical cable. The controller receives and processes the signals sent by the attitude sensors on the leg structure and sends the signals to the actuator to control the motions of the knee joint drive unit. The hip flexible strap serves to bridge the waist belt and the leg structure. The leg structure consists of a thigh structure, a shank structure, and a wrap system (i.e., thigh wrap and calf wrap). The actuator is located outside the knee joint, coaxial with the inner rotation axis of the leg structure, and roughly the same position as the rotation axis of the human knee joint flexion and extension movement. The rotational torque produced by the actuator is transmitted to the thigh and shank through the leg structure to provide additional flexion and extension power assistance of knee joint, reduce energy consumption of knee joint muscles, and enhance the wearer’s endurance.

#### Identify measurement trial tasks

**Figure 3:**
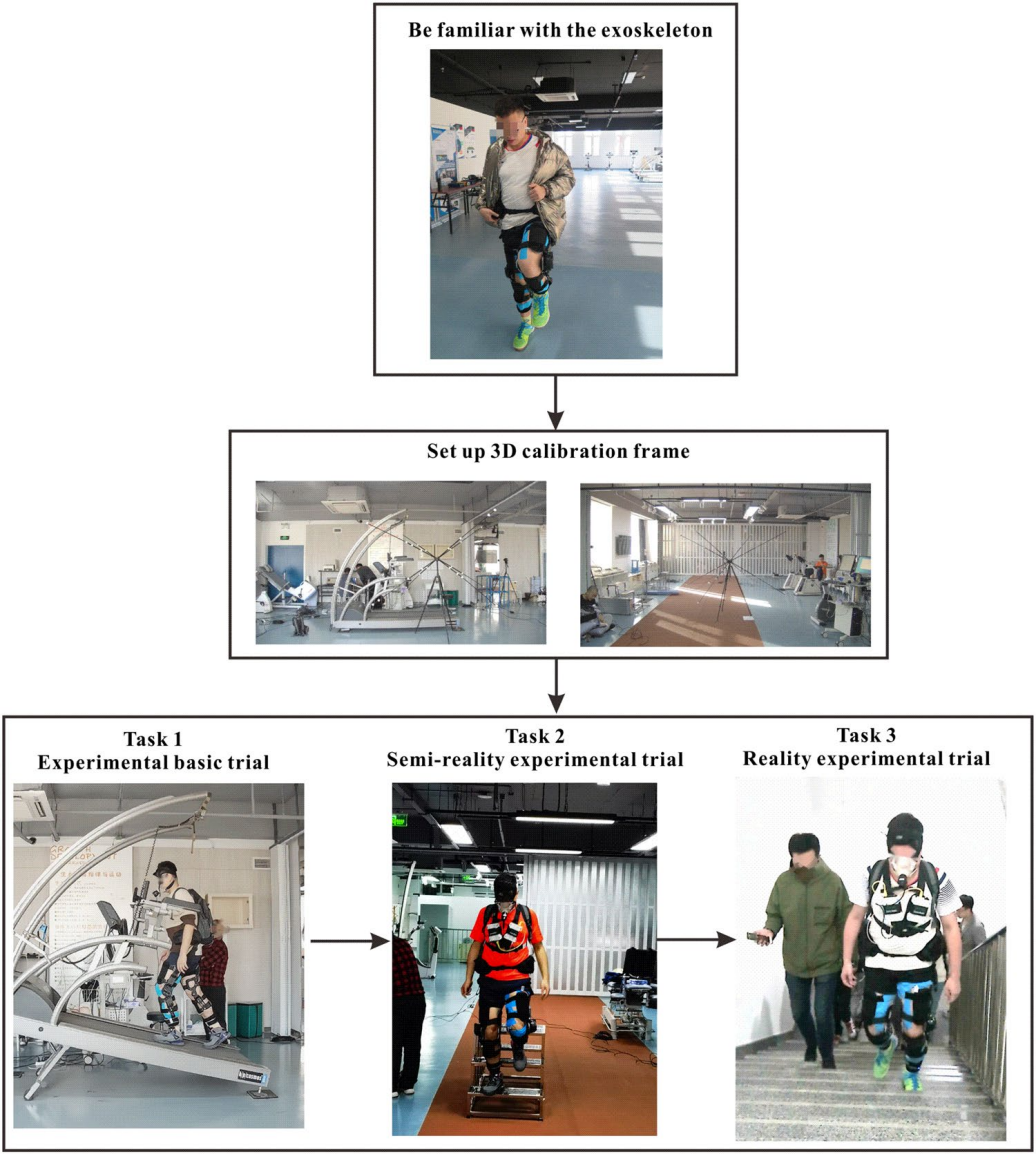
Performance test of the APAK exoskeleton. First, the subject should be familiar with the tested exoskeleton; second, a 3D calibration frame is set up. Finally, walking on treadmill at certain degree, walking up and down on three-step stairs, and ascending 11-storey stairs are carried out.

According to the test processing proposed by the IE approach, after the subject became familiar with the tested APAK exoskeleton, three trial tasks of measurement, including walking on a treadmill at certain angle, walking up and down on three-step stairs, and ascending in 11-storey stairs (Fig. 3), were formulated on the basis of fact applications and APAK exoskeleton scenarios to evaluate the following conditions: baseline, the subject walked without the exoskeleton; No assistance, the subject walked with the exoskeleton while the exoskeleton is operated in zero torque mode; Assistance, the subject walked with the exoskeleton operated in the assistance mode. The parameters of these trial tasks are shown in Table 1, and the nine aforementioned PIs are shown in Table 2.

**Table 1:** Parameters of trial task environments.

Motor skills	Parameters		
Walking on treadmill at certain degree	Incline angle	Speed	Duration
	14°	1.9 km/h	10 min
Walking up and down on three-step stairs	Height of a stair	Number of stairs	Duration
	22 cm	3	10 min
Ascending 11-storey stairs	Height of a stair	Number of each floor	Total height
	18–22 cm	14	35 m

**Table 2:** PIs characterized with three trial tasks. “√” indicates that the PI at that trial task was recorded, and “×” indicates that the PI at that trial task was not collected due to instrument limitations.

Performance indicators (PIs)	Trail tasks		
	Walking on treadmill	Walking on three-step stairs	Ascending 11-storey stairs
Goal-level			
Independence of donning/doffing process	**√**	**√**	**√**
Time of donning/doffing process	**√**	**√**	**√**
Task-based kinematics			
Joint angle	**√**	**√**	×
Joint angular acceleration	**√**	**√**	×
Human–robot interactions			
Metabolic cost			
HR	**√**	**√**	**√**
METs	**√**	**√**	**√**
RMS of sEMG	**√**	**√**	**√**
Muscle effort			
Average sEMG	**√**	**√**	**√**
Integral sEMG	**√**	**√**	**√**

These experimental trial tasks were conducted by one healthy subject (male, 30 years old, 1.80 m in height, 85 kg in weight) carrying 15 kg weights. These tests were based on the experimental protocol approved by the Ethics Committee of Capital University of Physical Education and Sports (Beijing, People’s Republic of China), and all experiments were performed in accordance with relevant guidelines and regulations. The subject provided written informed consent prior to the enrolment. Individuals identifiable in the included images gave their informed consent for publication.

#### Data collection and analysis

##### Goal-level

After the “start” command was given, the subject began to put on/take off the exoskeleton independently, and the tester started timing synchronously.

##### Task-based kinematics

Considering that outside ascending stairs was beyond the scope of optical motion capture system in our laboratory, the kinematic data were collected at the preliminary and intermediate phases. Four cameras were set up around the test region, and a 3D radial calibration frame (WFS-28 DLT Calibration Frame, HuiAnMing Sciences, Co., Ltd., Beijing, China) was set up and filmed for camera calibration.

##### Metabolic cost

The measured physiological variables include HR, *VO*_*2*_ [ml/min], and carbon dioxide production (*VCO*_*2*_ [l/s]). METs, *VO*_*2*_, and *VCO*_*2*_ were measured using a portable metabolic cart (K4 b^2^, Cosmed, Italy), whereas HR was measured using a mobile monitor (Polar’s HR sensors). HR value was calculated by averaging the value at the stationary stage of the HR graph. MET was calculated from VO_2_ so that 1 MET = 3.5 mlO_2_/kg/min^13^.

##### Muscle effort

The muscle activation during the entire cycle was measured by wired sEMG electrodes placed on RF and BF muscles of the subject’s dominant leg (left leg) using a 16-channel EMG system (YiShi KangLian Technology Co., Ltd, Shanghai, China) with a sampling frequency of 1000 Hz.

Maximum voluntary contraction (MVC) exercises were performed for each muscle to normalize EMG signals. EMG signals were integrated by a bandpass filter that ranges from 20 to 450 Hz and then processed with full-wave rectification, linearly enveloped, and finally normalized by MVC. The RMS of sEMG was defined with:

$$\mathrm{RMS}=\sqrt{\frac{{\sum }_{\mathrm{i}=1}^{\mathrm{N}}{\mathrm{x}}_{\mathrm{i}}^{2}}{\mathrm{N}}}$$

where N was window length for RMS calculation and taken as 10 in this study.

## Results

To be familiar with the exoskeleton suggested in the IE approach, the subject performed donning and doffing processes independently three times. The average time of completing donning/doffing processes was 67 s and 21 s, respectively, thereby indicating that APAK exoskeleton has user-friendly experience.

### Basic trial task

The task-based kinematics and human–robot interaction data of the subject who walked on treadmill at certain angle (1) with powered exoskeleton, (2) unpowered exoskeleton; and (3) without the exoskeleton were collected.

**Figure 4:**
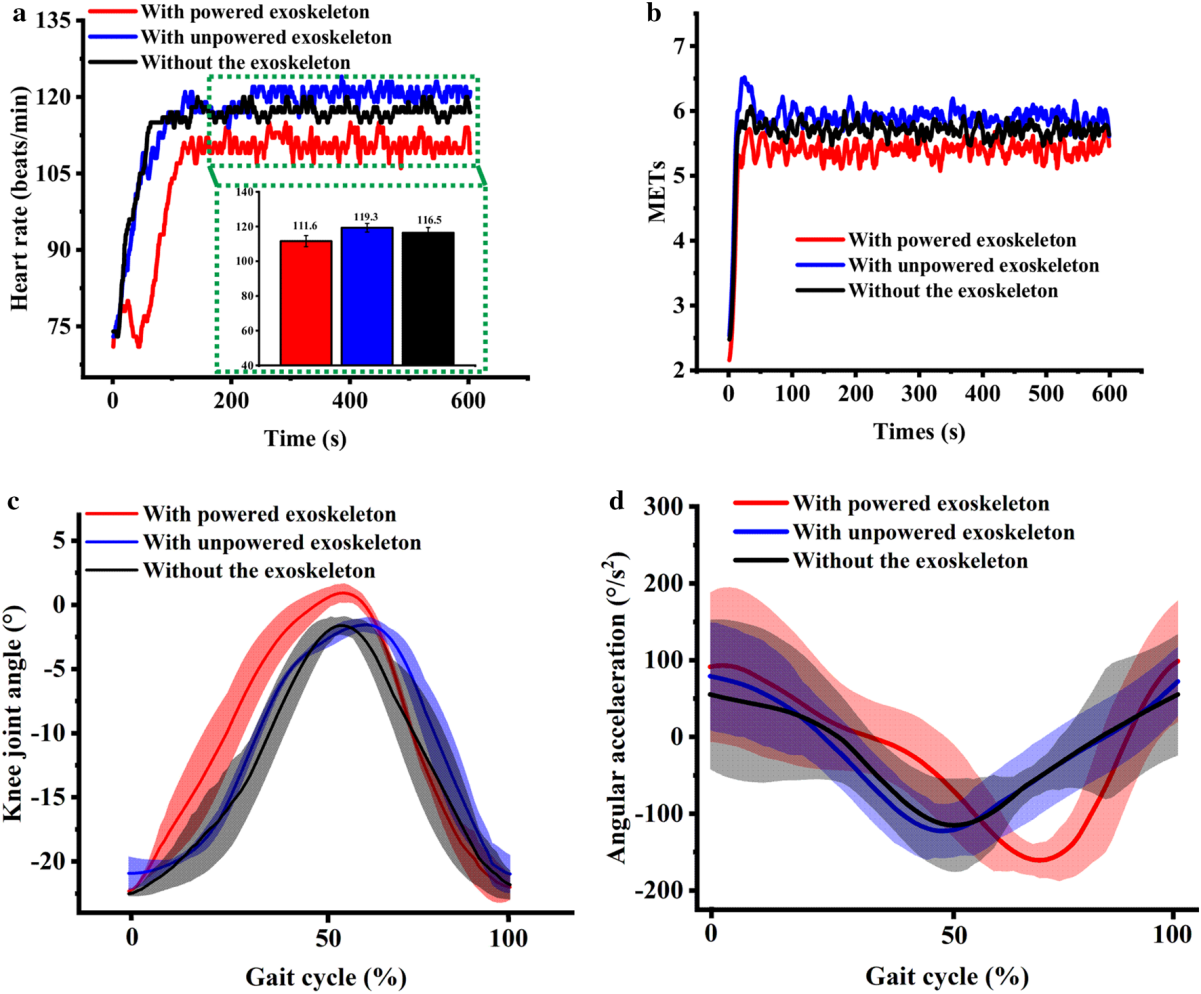
Kinematics and metabolic cost data of the subject with three conditions at the preliminary phase. (**a**) Heart rate value of the subject with three conditions, the average HR were inserted in green box; (**b**) MET changes in the subject with time during three conditions; (**c**) knee joint angle varies with time during two gait cycles; and (**d**) knee joint angular acceleration varies with time during two gait cycles, the shaded area represent the standard deviation.

The HR value at the stable stage in each instance is shown in Fig. 4a. The result shows that the HR value with “powered exoskeleton” is the smallest among the three conditions. The METs with powered exoskeleton were generally lesser than those in other conditions (Fig. 4b), indicating that the metabolic cost of the subject “with powered exoskeleton” will be reduced comparing with those “with unpowered exoskeleton” and “without exoskeleton”.

Figure 4c,d show the knee joint angle and angular acceleration of the subject in three conditions, in which negative angle value represents the knee joint flexion. The range of knee joint motions with exoskeleton, regardless of whether it is powered or unpowered, is similar for “without the exoskeleton”, indicating that wearing the exoskeleton does not restrict the activities of the knee joint. The peak of the knee joint angular acceleration “with powered exoskeleton” is larger than that “without the exoskeleton,” which is caused by the power-assisted performance of the exoskeleton.

**Figure 5:**
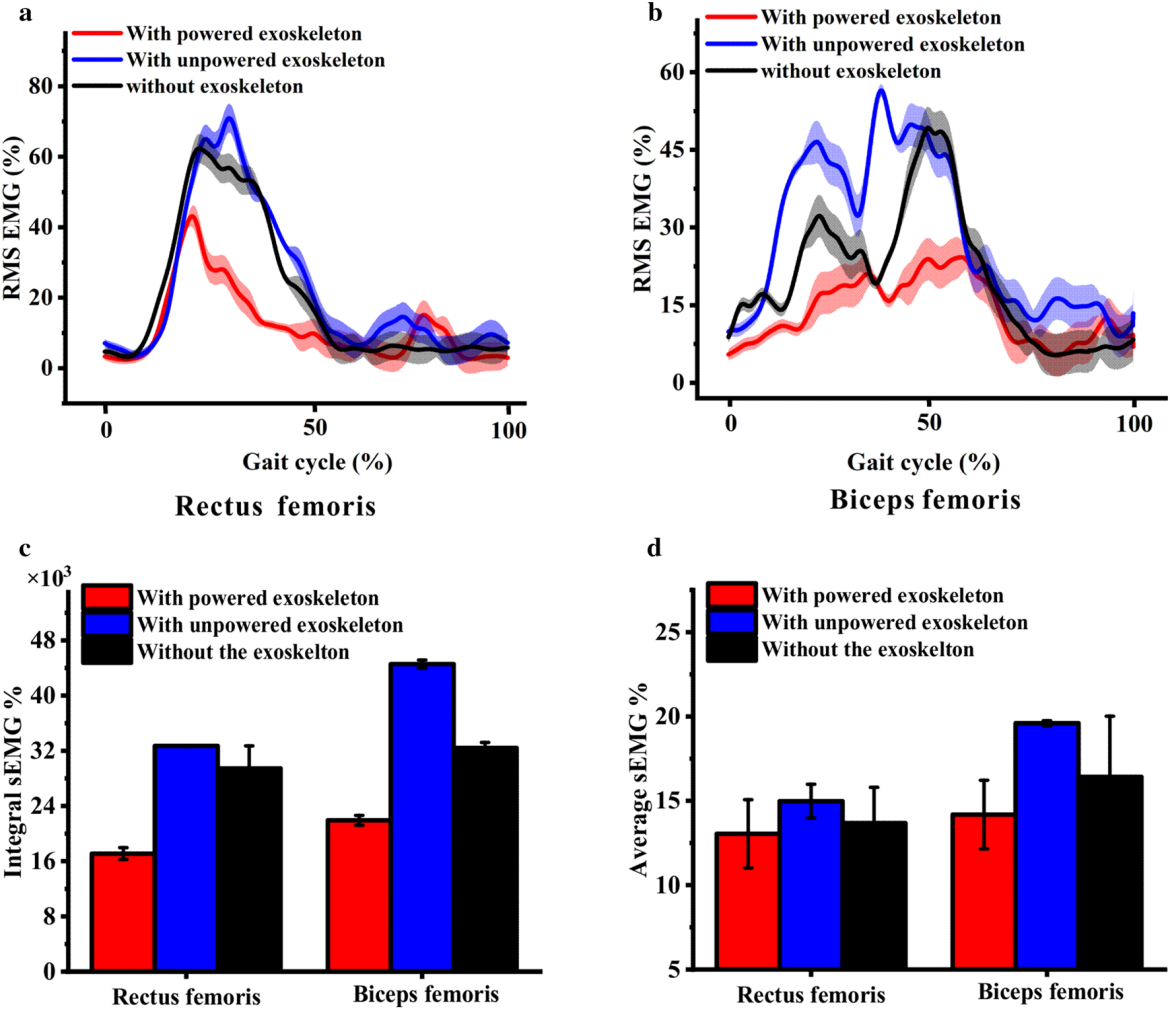
Surface EMG signals of BF and RF muscles during walking on a treadmill with a gait cycles. (**a**) RMS sEMG of RF with three conditions; (**b**) RMS sEMG of BF with three conditions; (**c**) IEMG of RF and BF muscles with three conditions; and (**d**) AEMG of RF and BF muscles with three conditions. The shaded area represent the standard deviation.

The sEMG signals of the RF and BF muscles are gathered during locomotion and compared with the results under the three conditions (Fig. 5). The RMS sEMG values of BF and RF muscles show a significant decrease in the “with powered exoskeleton” (Fig. 5a,b). The average EMG (AEMG) and integrated EMG (IEMG) values of these two muscles, which represent the overall muscle effort during two walking cycles, are smaller than those of other conditions (Fig. 5c,d). Hence, these results are consistent with that of root mean square (RMS) of sEMG.

According to the analysis results of task-related kinematics and human–robot interactions, we conclude that the rotational torque provided by electrical motors has possibly assisted the lower limb muscles during the performance augmentation. According to the IE approach, semi-reality trial could be conducted in the following step.

### Semi-reality trial task

In this section, the kinematics data and human–robot interaction data during walking up and down on three-step stairs with three conditions were recorded and calculated.

**Figure 6:**
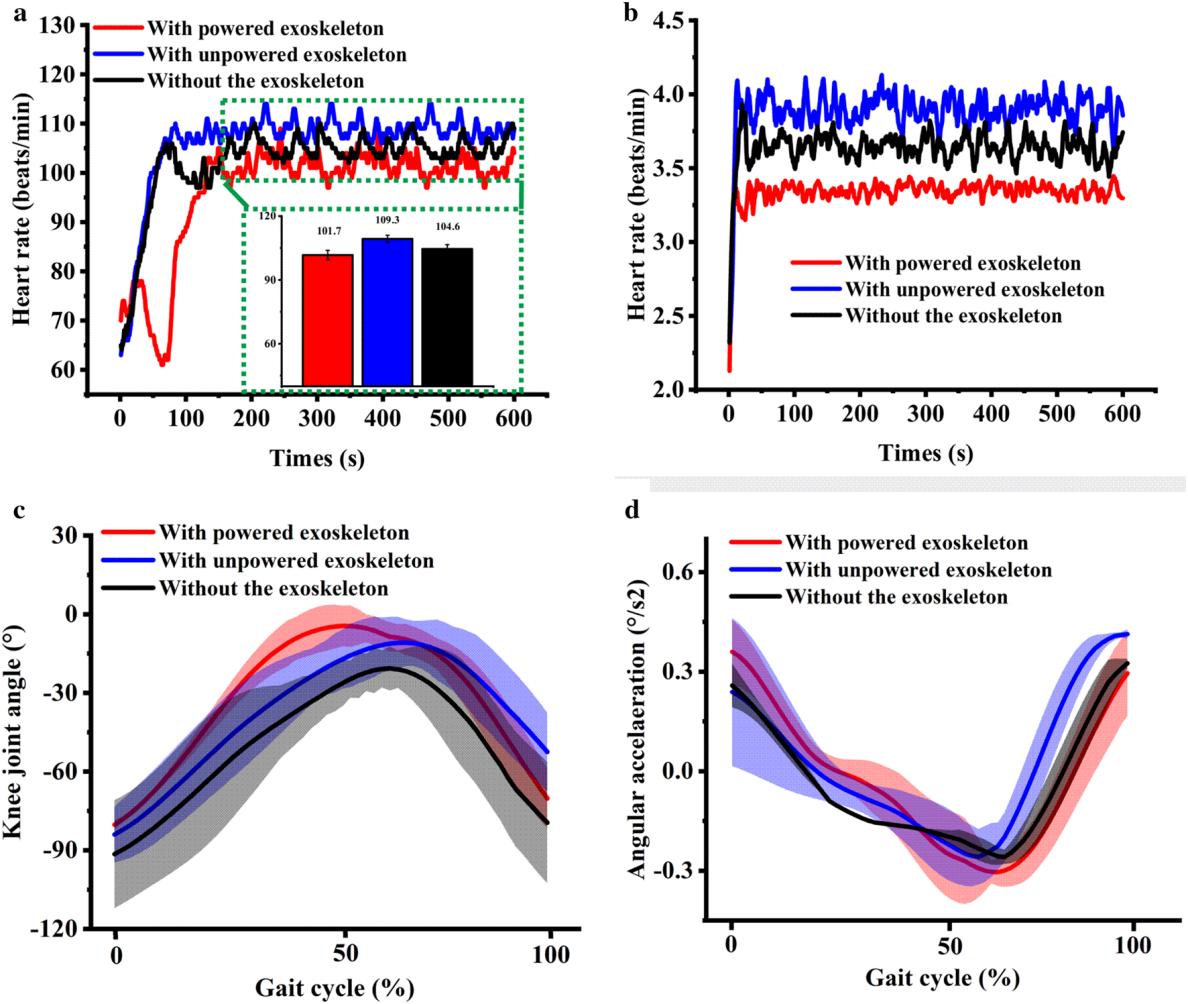
Kinematics and metabolic cost data of the subject with three conditions at the intermediate phase. (**a**) Heart rate value of the subject with three conditions, the average HR were inserted in green box; (**b**) MET changes in the subject with time during three conditions; (**c**) knee joint angle varies with time while walking upstairs; and (**d**) knee joint angular acceleration varies with time while walking upstairs, the shaded area represent the standard deviation.

The HR values and METs of the subject “with powered exoskeleton” are generally smaller than those in other conditions, which are similar for the result in Sect.  3.1 Experimental basic task, as shown in Fig. 6a,b. The kinematics data of walking upstairs are analyzed statistically. The results demonstrate that the range of the knee joint motion of the subject with exoskeleton is slightly restricted in an irregular terrain compared with that “without the exoskeleton” (Fig. 6c). The peak of the knee joint angular acceleration “with the powered exoskeleton” is larger than those in other conditions (Fig. 6d).

**Figure 7:**
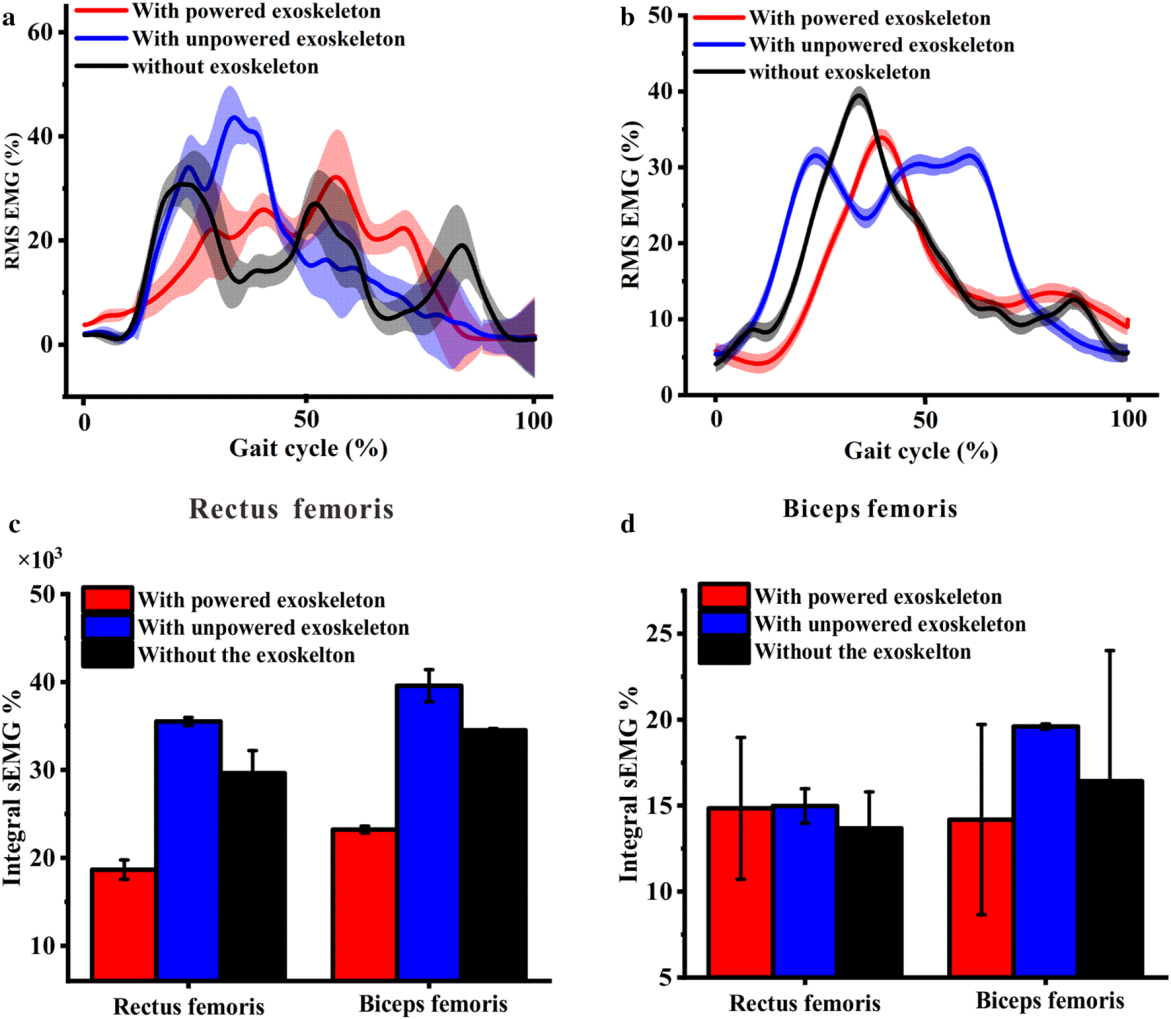
Surface EMG signals of BF and RF muscles during walking up stairs. (**a**) RMS sEMG of RF with three conditions; and (**b**) RMS sEMG of BF with three conditions, the shaded area represent the standard deviation; (**c**) IEMG of RF and BF muscles with three conditions; and (**d**) AEMG of RF and BF muscles with three conditions.

The RMS sEMG of RF and BF during two gait cycles are shown in Fig. 7a,b, respectively. The RMS sEMG of the RF and BF muscles of the subject “with powered exoskeleton” are generally lesser than those in other conditions, which are consistent with those in the basic trial task. The AEMG and IEMG of the RF and BF muscles also have a drastic decrease compared with the two other conditions, as shown in Fig. 7c,d.

Therefore, the APAK exoskeleton is considered to augment performance during the semi-reality trial. Starting the reality environmental trial task at the advanced phase is suggested.

### Reality trial task

**Figure 8:**
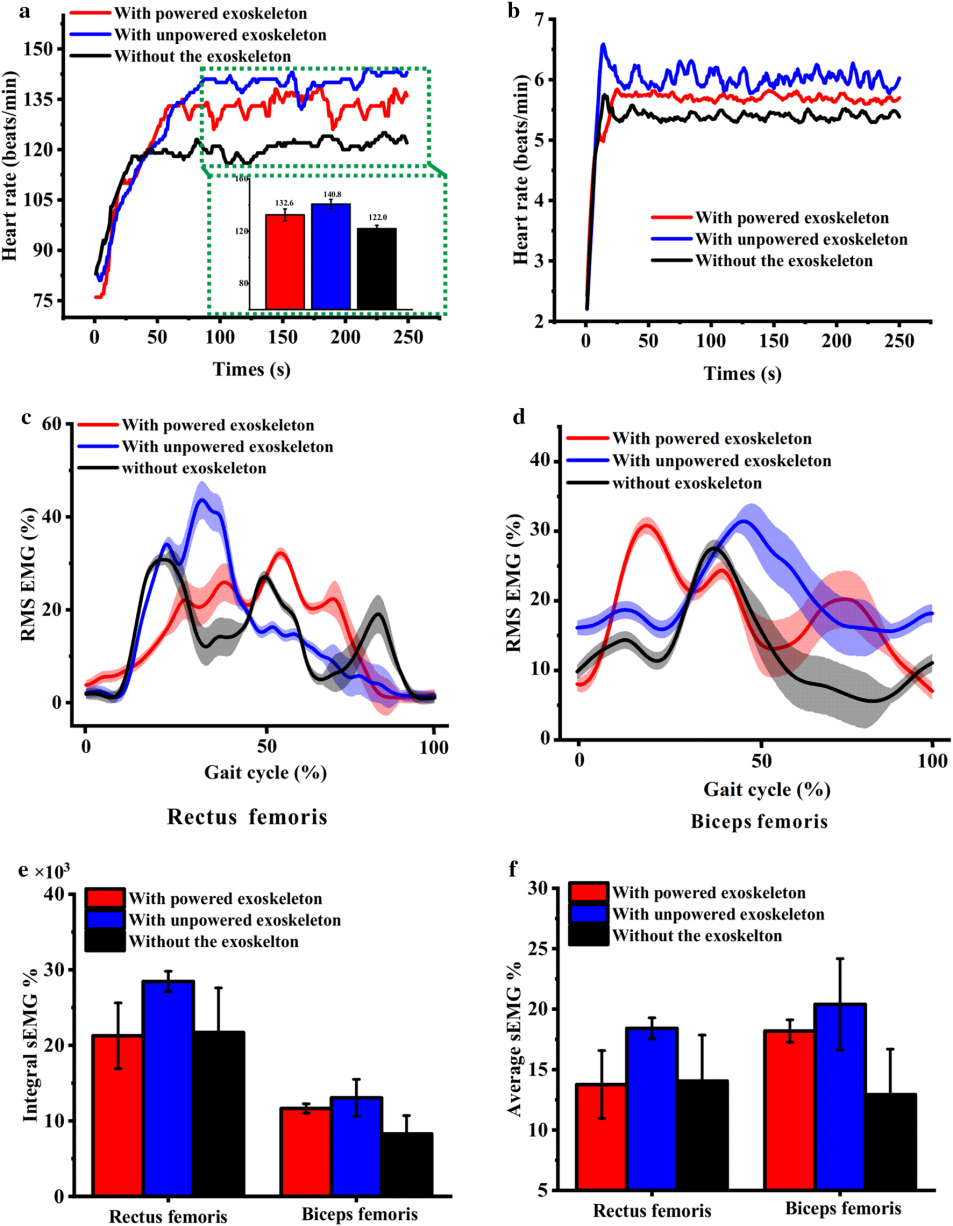
Kinematics and human–robot interaction data of the subject with three conditions at the advanced phase. (**a**) Heart rate value of the subject with three conditions, the average HR were inserted in green box; (**b**) MET changes in the subject with time during three conditions; (**c**) RMS sEMG of RF with three conditions; and (**d**) RMS sEMG of BF with three conditions, the shaded area represent the standard deviation. (**e**) IEMG of RF and BF muscles with three conditions; and (**f**) AEMG of RF and BF muscles with three conditions.

In this section, ascending 11-storey stairs outdoor was conducted. The human–robot interaction data are shown in Fig. 8. The HR value of the subject “with powered exoskeleton” is lower than that “with unpowered exoskeleton” and larger than that “without the exoskeleton,” as shown in Fig. 8a. The MET results show no significant difference between “with the exoskeleton” and “without the exoskeleton” (Fig. 8b).

The peak value of RMS sEMG of the RF muscle “with powered exoskeleton” is smaller than those in other conditions. However, the RMS sEMG of the BF muscle “with powered exoskeleton” has an opposite result, as shown in Fig. 8c,d, respectively. AEMG and IEMG results show that the peak value of the BF muscle “with powered exoskeleton” is significantly larger than that “without the exoskeleton,” which indicate that the APAK exoskeleton does not provide enough rotational torque to offset its own load exerted on the human body, resulting in the energy consumption increasing and local muscle activation, as shown in Fig. 8e,f.

Considering minimal performance augmentation of APAK exoskeleton in this reality trial task, it is suggested that the APAK exoskeleton is not suitable for marketing and should be further improved.

## Discussion

In this work, we have preliminarily developed a IE approach for assessing the power-assisted efficiency of lower limb exoskeleton that is mainly used in military or industrial fields given nine essential PIs that can characterize multiple aspects of human augmentation. An active power-assisted knee exoskeleton was used to assess its power-assisted effectiveness to verify the feasibility of the IE approach.

As we know, different kinds of lower limb exoskeletons have different criteria for judging “good” performance. For example, in the gait rehabilitation contexts, the clinical effect was considered to be prevail. Metabolic cost or usability indicators may dominate in military or industrial areas. When assisting paralyzed patients, stability and robustness are considered to be the most important content^16^. Thus, evaluating the power-assisted performance is extremely important to formulate an augmented-related trial task.

In the present work, three kinds of trial tasks with different difficulty, which closely related to the functions of lower limb exoskeletons in military or industrial areas, are established. The basic trial task is the simplest form of real environment in the laboratory. This trial task is usually completed only with a single action, such as walking on the level or inclined treadmill. Once all the recorded PIs demonstrate that the tested exoskeleton plays a power-assisted role during the test, the semi-reality trial task at intermediate phase can be launched. In the intermediate phase, the semi-reality trial task was constructed on the basis of simplifying an irregular terrain in real world. Similarly, the lower limb exoskeleton has provided enough assistance to augment performance during semi-reality trial task, thus reality trial task at advance phase could be carried out. The selection of real environmental terrains should be consistent with these power-assisted scenarios that are designed initially for lower limb exoskeletons. The replacement of a portable motion capture system was suggested considering that the kinematic data collections of outdoor trial task exceeds the scope of the optical motion capture system.

Nine essential PIs for evaluating the power assistance of lower limb exoskeletons are proposed and clustered into three aspects: goal-level, task-based kinematics, and human–robot interactions. The independence and time of donning and doffing processes for tested exoskeletons are considered critical for user-friendly experiences in the goal-level aspect. From the perspective of task-based kinematics, joint angle and joint angular acceleration were mainly used to compare the effects of “without the exoskeleton” on the joint rotation range, motion posture with “the unpowered/powered exoskeleton” of the subjects. In the frame of human–robot interactions, the metabolic cost was revealed by HR and METs, which could be used to demonstrate the total energy consumption of the subject. RMS of sEMG, AEMG, and IEMG were suggested to characterize the local antagonist muscle activation of the subject. During the basic trial and semi-reality trial tasks, the motion track of the marker points attached to the key joints is obtained through the motion capture system. The knee joint angle is determined by the spatial position of the ankle joint, knee joint and hip joint. The angular acceleration is determined by the second-order difference of the knee angular. Since these trial tasks is carried out on the sloping treadmill or 3-steps stairs, the knee joint angle gradually decreases during the whole support period of the gait cycle. In detail, the knee joint angle is largest at the early support period and smallest at the end.

The IE approach is being improved through continuous exoskeleton tests, and other useful PIs will be supplemented. For instance, the wrap system of the exoskeleton, which is directly contacted with the lower limb, is an extremely important bridge in the process of force transmission. Although the force transmission efficiency of the tight strap system is high, it may limit movement of the wearers. Nevertheless, the loose strap system of the exoskeleton has minimal obvious effects on the lower limb. Also, a PI related to the strap system of the exoskeleton must be proposed to characterize the efficiency of exoskeleton assistance. Furthermore, many times tests with different subjects in each instance are suggested to be conducted at one phase, once the nine PIs characterize the augmented performance, the next phase could be started. In addition, considering that subjects may adjust their stride frequency to keep pace with metronome and result in inaccurate evaluation of the tested lower-limb exoskeleton, it is not recommended to use metronome in the test tasks.

In conclusion, an IE approach for assessing the power-assisted efficiency of lower limb exoskeleton that is mainly used in military or industrial fields given nine essential PIs that can characterize multiple aspects of human augmentation. In the future, we will increase muscle synergy analysis to explore the effect of lower limb exoskeleton on human coordination and gait cycle; Increase the number of subjects to verify feasibility of the IE approach and furtherly assist to optimize the lower-limb exoskeleton. In addition, this approach could be modified adaptively to be applicable for evaluating upper limb exoskeletons. With the continuous improvement of the IE approach, it can be taken as a unified and broadly applicable benchmarking scheme for the performance evaluation of wearable lower limb exoskeletons and drive the wearable robotic community to demonstrate that our robots can meet real market needs.

## Data Availability

All data generated or analysed during this study are included in this published article.
